# COVID-19 and Diabetes: The Importance of Controlling RAGE

**DOI:** 10.3389/fendo.2020.00526

**Published:** 2020-07-14

**Authors:** Ernestina M. De Francesco, Veronica Vella, Antonino Belfiore

**Affiliations:** Department of Clinical and Experimental Medicine, University of Catania, and ARNAS Garibaldi, P.O. Garibaldi-Nesima, Catania, Italy

**Keywords:** COVID-19, diabetes, RAGE (receptor for advanced glycation end products), HMGB-1 (high mobility group box 1), pneumonia, coagulation disorders

## Introduction

The recent looming pandemic of Coronavirus disease 2019 (COVID-19) is caused by the novel severe acute respiratory syndrome coronavirus 2 (SARS-CoV-2), which has 80% homology with SARS-CoV-1 and 50% homology with the Middle East respiratory syndrome (MERS) viruses, both pathogens most likely originating from bats (1). While up to 80% of COVID-19 patients experience mild symptoms or remain asymptomatic, ~20% of them develop pneumonia that in nearly one third of cases presents as acute respiratory distress syndrome (ARDS) leading to severe hypoxia and possibly death (1). The risk of ARDS and mortality are increased in the presence of concomitant comorbidities like diabetes mellitus (DM). Herein, we propose that the Receptor for Advanced Glycation End Products (RAGE) and its ligands may play a pivotal role in COVID-19 pneumonia and ARDS, particularly in DM patients. While this paper was in preparation others have hypothesized a role for RAGE axis in COVID-19 pathogenesis and lung inflammation (2–4). In this opinion article, we extend these studies, and propose that targeting RAGE signaling system may hold potential in the clinical management of COVID-19 DM patients.

## Pathogenesis of COVID-19 Related Mortality

In the majority of patients, a finely tuned, and spatio-temporally coordinated response of both local innate and systemic adaptive immunity effectively clears SARS-CoV-2-infection. However, the cytopathic effect of the virus at the level of alveolar cells and vascular endothelium may induce massive pyroptosis, an inflammatory form of cell death linked to caspase 1 dependent activation of proinflammatory cytokines IL-1β and IL-18 by the NLR family pyrin domain containing 3 (NLRP3) inflammasome (5). In a minority of patients, a dysfunctional immune response occurs, leading to ARDS, multiorgan failure, and death, anticipated by the massive release of interleukin-17 (IL17), IL22, IL6, tumor necrosis factor-α (TNF), and other cytokines/chemokines. This so-called cytokine storm is associated with unbalanced immune responses including lymphocytopenia, impaired T cell function and deregulated Th17 cells differentiation, which leads to enhanced recruitment and activity of neutrophils and macrophages (1). In particular, IL6 tends to progressively increase in patients with severe ARDS; these patients may benefit from the treatment with anti-IL6 antibodies, such as Tocilizumab (6). Notably, an important component of ARDS is a lung-centric intravascular coagulopathy, which may evolve to multiorgan dysfunction with impaired microcirculatory function, thrombotic manifestations, and more rarely to disseminated intravascular coagulation (7, 8). Consistently, anticoagulation therapy with low molecular weight heparins (LMWH) is associated with decreased mortality in these patients. However, LMWHs may not be sufficient to revert pulmonary intravascular coagulopathy (7, 8).

On the basis of these observations, an unopposed inflammatory response mediated by hyperactivated immune effectors may play a key pathogenic role in ARDS of COVID-19 patients.

When it comes to unwarranted host immune response of COVID-19, lessons from studying bats are to be learnt. Bats are unique natural hosts for a number of RNA viruses with high pathogenic potential for humans, including SARS-CoV-1, and MERS related coronavirus. Notably, bats' ability to host these pathogens showing minimal or no signs of disease seems to be associated with a peculiar enhancement of innate immunity provided by constitutive expression of IFN-α and IFN-stimulated genes (9). Moreover, bats' extraordinary lifespan and viral tolerance, which seem to have evolved as protective mechanisms against flight-induced metabolic stress, appear to be related to a better overall execution of anti-inflammatory responses. Indeed, after viral infection, bats dampen excessive inflammation associated with the production of IL1β and IL18 by the NLRP3 inflammasome (10). Therefore, the enriched and highly competent innate immunity of bats halts the spread of pathogens and simultaneously freezes inflammatory pathways, thereby permitting a better control of infectious diseases.

Bringing this relevant piece of information back to the current pandemic might be useful to elaborate a therapeutic strategy in order to cope more efficiently with the severe clinical manifestations of COVID-19.

## Diabetes Mellitus As Risk Factor For COVID-19 Mortality

In COVID-19 patients, the development of severe ARDS is associated with advanced age, hypertension, severe obesity, and DM (1). In particular, a recent metanalysis indicates that DM significantly increases the risk of Intensive Care Unit (ICU) admission (OR: 2.79) as well as mortality (OR: 3.21) (11). The majority of DM patients suffer from Type 2 DM (T2DM) which is typically associated with obesity, insulin resistance, and multiple alterations of both the innate and adaptive immune responses (12, 13). Corroborating the role of dysregulated innate immunity in the progression of T2DM, peripheral blood mononuclear cells from diabetic patients have increased Th17 cytokine production, owing to dysregulated fatty acid oxidation occurring in these patients (14).

Moreover, T2DM patients are affected by chronic low-grade inflammation and endothelial dysfunction characterized by increased vessel permeability, and enhanced thrombotic propensity (15), these inflammatory-driven conditions are persistently supported by chronic hyperglycemia.

In this intricate scenario, RAGE, and its ligands have been involved in weight gain, insulin resistance, poor glycemic control, and inflammation, contributing to T2DM progression. Moreover, this complex signaling system does play a pivotal role in innate immunity, thereby representing one the first barriers against pathogens. However, unrestrained RAGE signaling supports and propagates inflammation thereby triggering tissue damage.

## Rage and its Ligands As Key Regulators of The Innate Immunity and Inflammation and Their Role in DM

RAGE has been named for its ability to bind advanced glycation end products (AGEs) that are found at increased levels in patients with hyperglycemia and contribute to chronic vascular complications of DM patients (16). In fact, AGEs-mediated RAGE activation is a major trigger of chronic endothelial dysfunction characterized by vascular hyper-permeability, increased of leucocytes adhesion, extravasation, and consequent acquisition of procoagulant status (17).

Moreover, RAGE is a transmembrane pattern recognition receptor (PPR) and a critical component of the innate immune system with the remarkable ability to bind numerous ligands including exogenous pathogen-associated molecular patterns (PAMPs), and danger-associated molecular patterns (DAMPs) released by cells undergoing damage or death (18).

Notably, RAGE is constitutively expressed at high levels only in the lung, at the basal membrane of type 1 alveolar epithelial cells (19), where it may contribute to cell adhesion and morphology that are functional to gas exchange. Its expression in type 2 alveolar epithelial cells is more controversial. In other cells, including endothelial cells, airway smooth muscle cells, vascular smooth muscle cells, neurons, and immune cells, RAGE expression is induced by inflammation and by local expression of RAGE ligands in a feed-forward loop that perpetuates inflammation. In the absence of ligands, RAGE may preassemble in multimeric complexes that are a prerequisite for activation and that are stabilized by ligand binding (20, 21). In DM patients RAGE is upregulated and chronically activated by AGEs (16).

**Figure 1:**
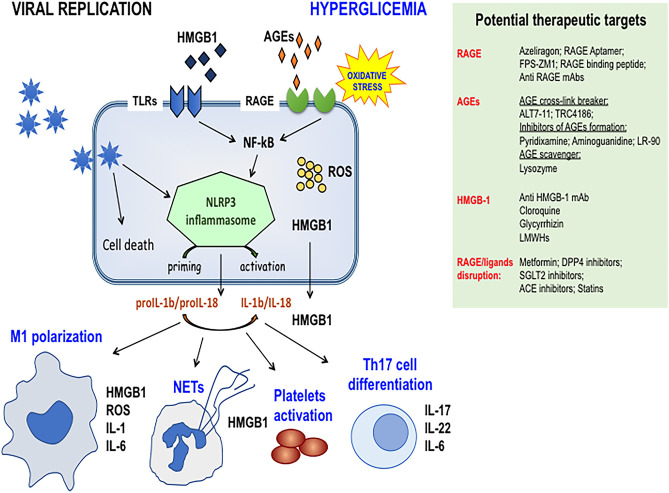
Schematic representation of the activation of RAGE axis in Sars-Cov-2 infected cells in DM patients. Hyperglycemia enhances the production of RAGE ligands that prompt viral replication and increase RAGE signaling activation thus contributing to NRLP3 inflammasome priming and activation. Cytokine production and HMGB1 passive secretion contribute to dysregulated immune cell responses like M1 macrophages polarization, NETs formation, altered Th17 lymphocytes differentiation, thus paving the way for the cytokine storm. The preexisting endothelial dysfunction of DM patients and the ability of HMGB1 to activate platelets contribute to the pathogenesis of lung-centric and eventually multiorgan intravascular coagulopathy. Side panel shows druggable targets of molecular and clinical intervention. Targeting the RAGE pathway may represent a useful and feasible strategy for controlling severe ARDS and coagulopathy, particularly in DM patients. DPP4, Dipeptidyl-peptidase 4; SGLT2, Sodium-Glucose Cotransporter 2; LMWH, Low Molecular Weights Heparins.

Besides AGEs, RAGE binds also several other ligands (Figure 1), among which protein HMGB1 (high mobility group box 1) and S100/calgranulin proteins have been extensively characterized. HMGB1 and S100 share the ability to be passively released from damaged cells and to be actively secreted by immune cells, such as macrophages, natural killer cells, and dendritic cells. Both classes of proteins contribute to the inflammatory response by binding to RAGE and members of the Toll-like Receptors (TLRs) family.

In particular, RAGE activation triggers the NF-κβ-mediated transcription of inflammatory genes, together with the activation of the NRP3 inflammasome. These events may ultimately lead to pyroptosis, which determines the release of further mediators from dying cells and the propagation of the initial damage (10, 22).

## Rage and Ligands in Pneumonia and ARDS

Direct evidence that RAGE may play a detrimental role in pathogen-induced pneumonia in the context of DM come by recent studies assessing that diabetic mice challenged with Gram-negative bacteria (GNB) show excess mortality from pneumonia (23) that was associated with RAGE mediated hyperinflammation. Accordingly, RAGE blockade improved survival of only diabetic mice during GNB infection (23). Moreover, accumulating evidence suggest that RAGE and its ligands play a significant role also in viral pneumonia and in ARDS.

HMGB1 is also upregulated by hyperglycemia and appears to play a major role in lung inflammation especially in the context of DM. In a mouse model of DM, HMGB1 was found to maintain lung inflammation through the RAGE/AKT1/β-catenin pathway (24). Moreover, HMGB1 is implicated in deregulated differentiation of Th17 cells and, by binding to RAGE, increases the expression of NLRP3 inflammasome components, and of IL1β (25).

In pneumonia and ARDS, HMGB1 is emerging as a biomarker of mortality risk, whereas in child pneumonia HMGB1 permits to discriminate between coinfection (bacterial and viral) vs. single infection. Adding pieces to this puzzle, circulating levels of soluble RAGE (sRAGE, a decoy receptor) positively correlate with ARDS severity and mortality risk, whilst sRAGE drop is associated with disease resolution (18, 19). Therefore, monitoring the levels of sRAGE and ligands may represent an appropriate tool for predictive/prognostic purposes.

Notably, RAGE, HMGB1, and S100 proteins have all been involved in coagulation disorders. In particular, HMGB1 directly stimulates platelets through TLR4, and RAGE, while S100 proteins released from neutrophils and platelets facilitate thrombus formation through RAGE activation (26). In turn, vascular damage induces huge release of HMGB1 from platelets (27). Moreover, HMGB1 and hyperglycemia prime neutrophils for the formation of NETs (Neutrophil Extracellular Traps), complex webs of chromatin and antimicrobial proteins released by neutrophils in an attempt to contain infections (28). However, unrestrained NETs release, which may especially occur in COVID-19 patients receiving mechanical ventilation (29), may contribute to the propagation of inflammation and the establishment of microvascular thrombosis (30). Reinforcing this self-perpetuating loop, NETs themselves may act as a source of HMGB1 (31), which enhances also vascular permeability (32). Of note, endothelial cells (EC) are a direct target of SARS-CoV-2 infection that induces a widespread endotheliitis characterized by acute ECs dysfunction/death and vascular leakage (33). Together, these observations suggest that chronic EC dysfunction of DM patients predispose to further EC damage by SARS-CoV-2 infection (34) and that RAGE is an important molecular hub in this context.

## Possible Role of Rage in SARS-COV-2 Cell Entry

Finally, there are evidence that, besides binding to the main receptor ACE2 (35), SARS-CoV-2 spike protein may bind also to CD147 glycoprotein (36), a multiligand protein that is upregulated by hyperglycemia and by RAGE activation (37). CD147 is highly expressed in type II pneumocytes and in a wide range of other cells, including immune cells, endothelial cells, and platelets. Interestingly, CD147 is involved in hyaluronan production, which has a key role in COVID-19 pneumonia and elicits pro-inflammatory and pro-thrombotic actions (38). AGEs—induced CD147 glycosylation in endothelial cells may increase the activity of metalloproteinases (MMPs) and loosen tight junctions. These findings raise the intriguing possibility that RAGE activation may play a role also in viral invasion to host cells.

## Therapeutical Perspectives

These considerations raise important questions concerning the best therapeutic management of COVID-19 DM patients. Recent evidence indicates that a tighter glucose control is associated with a better outcome and reduced mortality (39, 40). Further studies are actually needed to ascertain whether plasma levels of AGEs, sRAGE, HMGB1, and S100 proteins in DM COVID-19 patients are differentially affected by conventional vs. intensive insulin therapy and are predictive of patients' outcome. An encouragement to chase this track more in depth comes from the observation that in non-COVID-19 critically ill hyperglycemic patients admitted at ICU, plasma levels of sRAGE are higher in DM vs. non-DM patients, and reduced by intensive insulin therapy only in DM patients (41). In a similar series of patients, sRAGE levels were associated with circulatory and kidney failure and a higher rate of mortality but were not affected by insulin therapy (42).

Targeting RAGE axis to get a better control of COVID-19-related inflammation has been very recently proposed by independent investigators (2–4). We suggest that COVID-19 DM patients could be the subset most likely to get the highest therapeutic advantage from these strategies.

In this context, several pharmacological approaches are immediately available for controlling aberrant RAGE activation (Figure 1), although none of them has been purposely evaluated in patients with COVID-19 pneumonia and ARDS, with or without DM.

The small molecule azeliragon is an orally bioavailable and overall well-tolerated RAGE inhibitor that prevents the interactions with ligands. Additional FDA-approved drugs are known to disrupt RAGE signaling (Figure 1), thus suggesting that a drug repositioning effort could provide a fast track for controlling at least some of the negative outcomes associated with severe ARDS.

However, it should be noted that chloroquine, currently used in COVID-19 therapy, inhibits HMGB1 release especially in DM patients (43). In this last clinical setting, interfering with the HMGB-1/RAGE axis could serve as a further tool in combination strategies with IL-6 receptor mAbs, as HMGB-1/RAGE interaction contributes to IL6 expression (44).

Moreover, LMWHs, such as 2-O,3-O-desulfated heparin (ODSH), are able to inhibit interaction of RAGE with HMGB1 and S100 calgranulins (45). Enoxaparin may also elicit similar effects on HMGB1 (46). Accordingly, in experimental models the use of neutralizing anti-HMGB1 monoclonal antibody confers protection against lung injury and pneumonia, including pneumonia from influenza virus (47).

Additionally, thrombomodulin (TM) a protein expressed by the vascular endothelium that interacts with various proteins and inhibits coagulation and inflammation, has been shown to bind and block HMGB1 binding to RAGE (48). Administration of soluble recombinant TM (srTM) has shown some benefit in a mouse model of surgical ARDS via suppression of HMGB1 (49). In humans, administration of srTM significantly reduced 28-day mortality in patients with sepsis-associated coagulopathy (50).

Recently, human recombinant soluble (s)ACE2 has been shown to inhibit the early stages of SARS-CoV-2 infections (51). As ACE2 has a protective role in lung injury (52, 53) and it may downregulate HMGB1 (54), the use of sACE2 might be beneficial also at later stages of COVID-19.

## Discussion

Several mechanisms have been implicated for the increased risk of DM patients to develop severe COVID-19 disease (11). For the first time, we suggest that chronically activated RAGE/RAGE ligands axis, may be an additional and important mechanism. The RAGE system is a critical player in innate immunity and coagulation homeostasis, therefore, there is hope that targeting this system could halt the cytokine storm and the thrombotic manifestations associated with dysregulated immune responses to SARS-CoV-2 infection, especially in DM patients.

## Author Contributions

All authors collected and discussed the literature, with special participation of AB and VV for clinical aspects of Covid-19 and their relation with diabetes mellitus, of ED for the RAGE system and of AB for ARDS and viral pneumonia. VV and ED prepared the figure. AB wrote the manuscript which was substantially integrated, revised, and approved in its final version by all authors. All Authors contributed to the conceptualization and design of the review.

## Conflict of Interest

The authors declare that the research was conducted in the absence of any commercial or financial relationships that could be construed as a potential conflict of interest.
